# Materials, Processing, and Post-Treatment for Metal-Based Additive Manufacturing

**DOI:** 10.3390/ma18184311

**Published:** 2025-09-15

**Authors:** Liyuan Sheng, Junke Jiao, Hui Zhao

**Affiliations:** 1 Laboratory of Advanced Materials and Processing, Peking University Shenzhen Institute, Shenzhen 518057, China; 2 School of Mechanical Engineering, Yangzhou University, Yangzhou 225009, China; jiaojunke@yzu.edu.cn; 3 School of Material Science and Engineering, Xi’an Shiyou University, Xi’an 710065, China; huier7921@126.com

Recently, additive manufacturing (AM) has attracted significant attention due to its ability to fabricate components with irregular and complex shapes [1]. Its diverse fabrication techniques—such as powder bed fusion (PBF), directed energy deposition (DED), fused filament fabrication (FFF), materials extrusion (ME), binder jetting (BJ), and stereolithography (SLA)—offer a wider array of options for preparing different materials [2,3,4,5,6,7]. Furthermore, advancements in materials specifically designed for AM, as well as the hybridization of AM techniques, are expanding its applications across various fields, such as aerospace, transportation, automotive, marine equipment, and medical devices [8,9]. It is anticipated that AM will revolutionize materials fabrication.

Despite its numerous advantages, AM also faces several challenges. The rapid forming process can introduce significant internal stresses, resulting in the formation of microcracks [10]. In addition, the layered formation mode produces typical microstructural repetitions and interlayer interfaces, often producing uneven surfaces and relatively high roughness [11]. Compared to conventional material molding, many AM techniques reduce the time needed for melt or fluid redistribution, which can result in the formation of microvoids [12]. These characteristics may lead to various defects in AM-fabricated components, severely compromising their performance and service life [13,14]. In addition, adapting materials to different service environments necessitates surface modification, which is another important factor to consider.

To address these challenges and meet diverse application requirements, post-treatment processes are widely employed to optimize the microstructure and material properties of these components [15]. Given the relationship between material composition and fabrication processes, understanding the systematic correlation between post-treatment and AM-fabricated components is crucial. Therefore, it is essential to explore the distinctive characteristics of AM fabrication and its corresponding impacts. This Special Issue features ten research and review articles covering the following topics: fabricating components with complex structures by AM, surface modification, optimization of hybridized AM parameters for specific metals, enhancement of Ti6Al4V laser joint through laser shock peening, additive manufacturing of tungsten alloys for plasma-facing materials, and laser cladding on Ti and its alloys. The research papers present interesting findings that improve the performance of AM-fabricated components, while the review papers illustrate the current developments and future challenges in related fields.

Through the reports and discussions presented in these studies, we aim to draw greater attention to the critical field of metal-based AM. The focus lies in uncovering the complex relationships between materials, processing, and post-treatment in AM, which are key factors in enhancing the quality and performance of printed components. By showcasing recent advancements, we anticipate significant progress in developing improved materials, equipment, and procedures for manufacturing superior AM components. Furthermore, we encourage further research in this area and look forward to receiving more high-quality submissions for the next Special Issue.

The Guest Editors of this Special Issue extend their gratitude to the authors for their significant and high-quality contributions, the reviewers for dedicating their time and effort in improving the submissions, and the publisher for their outstanding work and collaboration.
